# Olive Oil in Cholelithiasis

**Published:** 1907-05

**Authors:** 


### SELECTIONS AND ABSTRACTS.

### OLIVE OIL IN CHOLELITHIASIS.

Dr. R. Alexander Bate, of Louisville, Kentucky, believes that olive-oil does dissolve fresh cholesterin calculi and that all conditions characterized by an excess of organic acids are accompanied by gall-stones; and states that only the bilirubin calcium calculi appear unchanged in the evacuations of those receiving olive-oil internally. Surgery, he thinks, is only indicated in cases where exhaustion would take place before solution of the calculi could occur. Of 14 patients observed by the author, all of which had had some medical treatment, three were operated on and died. In two instances a recurrence took place. Nine patients had been apparently cured by the medical treatment.—The Medical Bulletin.

